# From Adolescent Stress Mindset to Positive Behavior: The Moderation Role of Social Support and Sex Differences

**DOI:** 10.3390/bs16071060

**Published:** 2026-06-26

**Authors:** Xu Jiang, Shannon M. Testa, Marissa F. Mulvey

**Affiliations:** Department of Psychological Studies in Education, Temple University, 1301 Cecile B. Moore Ave., Philadelphia, PA 19122, USA

**Keywords:** mindset, implicit theory, stress mindset, social support, personal growth initiative, strengths use, adolescents

## Abstract

Based on the mindset × context framework, the interplay between individual mindsets and social context factors should be considered together while analyzing mindset’s effects on developmental outcomes. This study focuses on how stress mindsets might interact with social support to predict two positive behaviors (personal growth initiative and strengths use) in adolescents using a moderation model and whether a such mechanism differs across sex via a moderated moderation model. Participants were 620 high school students aged 14 to 19 years (M = 17.51, SD = 1.23), from diverse U.S. regions, who completed an online self-report survey in spring 2022. Statistical analyses were conducted using the PROCESS macro in SPSS. The results showed that the moderation effect in the single moderation model was not significant, while all interaction terms were statistically significant in the moderated moderation model. Specifically, the magnitude of the positive relation between stress mindset and strengths use was weaker at higher levels of social support among males; however, this relation was stronger when the social support level was high for females, showing the opposite trend. Overall, the results support the mindset × context framework and highlight the different mechanisms by which mindsets and social support work together between males and females.

## 1. Introduction

People’s beliefs about stress, or stress mindsets, are connected to psychological and behavioral processes and outcomes, such as coping and distress (Crum et al., 2013). However, most prior work has focused on associations between stress mindsets and individual-level factors, with limited attention to contextual factors that may shape the role of stress mindsets. Based on the mindset × context framework (Carroll et al., 2023), the effects of mindsets also depend on features of the social environment, which should be incorporated into research on understanding mindsets. In addition, little is known about how stress mindsets relate to positive behaviors such as personal growth initiative and strengths use, which reflect intentional self-development and the application of personal strengths, respectively. The present study addresses these gaps by examining whether perceived social support moderates the association between stress mindsets and these positive behavioral outcomes; the study also explores potential sex differences in these associations.

### 1.1. Stress Mindset

Mindsets refer to beliefs about the changeability of one’s personal characteristics and abilities, which influences how they perceive and respond to different situations. Those with “fixed” mindsets believe that abilities are static and innate, while those with “growth” mindsets believe that abilities can be developed over time through effort and perseverance (Yeager & Dweck, 2012). Individuals with growth mindsets are more likely to have adaptive outcomes compared to those with “fixed mindsets” (Yeager & Dweck, 2020). The concept of a stress mindset has emerged as a distinct type of mindset, referring to the extent to which an individual believes that stress has enhancing effects on outcomes such as productivity, learning, health, and well-being (Crum et al., 2013). Specifically, those with a “stress-is-enhancing” mindset tend to view stressful situations as beneficial and potential opportunities for growth, while those with a “stress-is-debilitating” mindset tend to view stressful situations as harmful and leading to negative outcomes (Park et al., 2018).

Stress mindsets are linked to the use of adaptive coping strategies. Adolescents who endorse a stress-is-enhancing mindset tend to engage in more adaptive forms of coping and hold stronger beliefs in their capacity to manage stress. For example, adolescents who endorsed a stress-is-enhancing mindset reported greater use of engagement coping strategies (e.g., help-seeking and problem-solving) during the COVID-19 pandemic, which was associated with higher well-being (Caleon et al., 2023). Similarly, adolescents with stress-is-enhancing mindsets were more likely to believe in their ability to handle stress and to view social stress as an opportunity for learning and improvement (Subhasree et al., 2023). Several studies also suggest that “stress-is-enhancing” mindsets may act as a buffer against psychological distress and maladaptive coping strategies when adolescents face adversity. Specifically, endorsement of the “stress-is-enhancing” mindset has been linked to a reduced biological response to stress, lower perceived stress and distress, improvement-oriented behaviors, and a decreased risk for depression when compared to those with a “stress-is-debilitating” mindset (Crum et al., 2013, 2017; Huebschmann & Sheets, 2020). These findings suggest that stress mindsets play a role in an individual’s responses to stress.

Emerging research on stress mindsets in adolescents has examined their associations with mental and behavioral health outcomes and their role as a protective factor. Studies across diverse samples suggest that endorsing a stress-is-enhancing mindset is linked to more favorable psychological outcomes. For example, students with stress-is-enhancing mindsets reported lower anxiety, depression, and stress before school exams and were more likely to view exams as a challenge rather than a threat (Wang et al., 2022), and students with stress-is-enhancing mindsets and higher academic buoyancy were less likely to experience academic burnout (Lakkavaara et al., 2024). Research with rural-to-urban migrant adolescents in China further indicates that stress-is-enhancing mindsets function as protective factors in the context of adversity: adolescents with stress-is-enhancing mindsets were less prone to depression when encountering stressful life events, and stress-is-enhancing mindsets, along with resilience, buffered the negative effects of the cumulative risk on emotional well-being (Y. Jiang et al., 2020). Additionally, stress-is-enhancing mindsets, combined with approach coping styles, reduced depression and the negative impacts of stress (Chen & Qu, 2021) and, together with beliefs in subjective social mobility, mitigated the effects of adverse circumstances (Ming et al., 2021). Evidence from other populations also suggests protective effects, including buffering against stress and impulsive behavior (Park et al., 2018), reducing intentions to use and the use of cannabis during the COVID-19 pandemic (Wilson et al., 2025), protecting against the impacts of academic expectation stress on online gaming disorder (Cheng et al., 2024), and moderating the relationship between family conflict and adolescent depression (S. Jiang et al., 2025). Together, these findings suggest that stress-is-enhancing mindsets are associated with greater use of adaptive coping strategies and both reduced negative mental health and behavioral outcomes during adolescence.

### 1.2. The “Mindset × Context” Framework

Much mindset research to date has focused on the individual level, examining how mindsets influence motivation, behavior, and academic outcomes. However, this individual-level focus does not fully capture the role of context in shaping how adolescents act on beliefs. The “mindset × context” framework proposes that the extent to which individuals act in accordance with their beliefs is shaped by the opportunities and constraints present in their social environments (Hecht et al., 2021). This theory suggests that beliefs alone are insufficient for shifting or changing behaviors if adolescents are in contexts that do not support or reinforce growth-oriented behaviors. The concept of psychological affordances, defined as opportunities within social contexts that permit particular ways of interpreting and responding to experiences, helps to explain how context interacts with mindsets to influence behavior (Walton & Yeager, 2020). For example, a growth mindset may only translate into persistent effort or adaptive coping if classrooms, families, and peers provide supportive feedback, model adaptive responses, and value learning from challenges. As such, without enough environmental supports, even with stress-is-enhancing mindsets, adolescents may still have difficulty in enacting growth-oriented behaviors.

This theory is still relatively new, with emerging evidence supporting it. For example, using data from the National Study of Learning Mindsets, Yeager et al. (2022) found that the effectiveness of a growth mindset intervention depended on teachers’ own mindsets; intervention effects were stronger when students’ growth mindsets were supported by more growth-oriented teacher contexts. Similarly, Bostwick et al. (2022) found that students’ growth orientation was associated with improvements in mathematics outcomes and that classroom-level growth orientation moderated the relation between individual students’ growth orientation and adaptive engagement. Together, these findings support the mindset × context framework in that the benefits of mindsets may depend on the surrounding social context, including classroom and teacher-level factors, which can either support or limit the translation of beliefs into adaptive behaviors. Both this theoretical framework and supporting evidence suggest that individuals’ mindsets and contextual factors should be considered in evaluating the role of stress mindsets in youth developmental outcomes.

### 1.3. Positive Behaviors

Beyond buffering negative outcomes, stress-is-enhancing mindsets may promote positive behaviors that reflect intentional engagement in growth and adaptive functioning. Adolescents who endorse stress-is-enhancing mindsets tend to interpret challenges as opportunities, persist in the face of difficulty, and engage in proactive coping strategies, such as problem-solving and help-seeking. For example, stress-is-enhancing mindsets have been associated with greater use of proactive coping, which was then associated with more positive challenge appraisals, highlighting the way in which these beliefs can foster growth-oriented behavior (Mansell & Turner, 2023). In addition, emerging research suggests that stress mindsets are malleable and can be modified to promote adaptive behaviors; for example, a cognitive–behavioral intervention conducted with adolescents demonstrated increases in stress-is-enhancing mindsets alongside improvements in proactive coping and psychological well-being (Sparks et al., 2025). These behaviors reflect a broader orientation toward self-directed development, including personal growth initiative and strengths use.

#### 1.3.1. Personal Growth Initiative

Personal growth initiative (PGI) refers to an individual’s active and intentional involvement in personal development and self-change (Robitschek, 1998). Rather than experiencing growth passively, adolescents high in PGI deliberately engage in behaviors aimed at self-improvement, including planning, goal-setting, and utilizing resources. Research has linked higher PGI to greater psychological well-being, lower distress, and more adaptive identity processes in adolescents and emerging adults (Ayub & Iqbal, 2012; Luyckx & Robitschek, 2014). However, little is known about factors that foster PGI in adolescence, and no studies to date have examined whether stress mindsets may serve as one such factor that encourages proactive engagement in personal growth.

#### 1.3.2. Strengths Use

Strengths use involves actively applying one’s personal strengths in daily life (Wood et al., 2011). While possessing strengths is valuable, the deliberate use of strengths has been associated with higher well-being, increased positive affect, and lower perceived stress (Wood et al., 2011). Research with adolescents suggests that contextual factors, such as strengths-based parenting, can enhance the use of strengths and therefore improve subjective well-being (Jach et al., 2018). Although early evidence indicates that adolescents with growth-oriented beliefs about strengths are more likely to use them, it remains unclear whether broader stress mindsets (specifically, viewing stress as enhancing) also encourage strengths use in everyday life.

Therefore, studying PGI and strengths use in addition to stress mindsets is critical in understanding the mechanisms through which adolescents translate beliefs into growth-oriented behaviors. There is a gap in knowledge regarding how stress mindsets might foster positive developmental behaviors. Exploring these associations can clarify mechanisms by which mindsets support coping and resilience, as well as intentional engagement in personal growth and strengths-based behaviors, ultimately contributing to adolescents’ psychological well-being.

### 1.4. Social Support

A critical component of context is social support, which has been conceptualized as the extent to which individuals perceive that they are cared for, valued, and part of a network of meaningful social relationships (Cobb, 1976). Social support plays a central role in adolescent development and engagement in positive behaviors; adolescents who perceive strong support from parents, peers, and teachers demonstrate greater resilience, self-efficacy, and adaptive coping, as well as lower levels of depression and psychological distress (Rueger et al., 2016). Supportive relationships provide encouragement, guidance, and feedback that help adolescents to navigate challenges, learn from experiences, and engage in adaptive behaviors. In particular, adolescents are more likely to pursue developmental goals, persist in difficult tasks, and apply personal strengths when they feel understood and supported by important adults and peers. These patterns highlight the importance of considering social support as a key factor in understanding how adolescents translate their beliefs and motivations into positive behaviors. In this study, we use social support as an indicator of the contextual factor to test the plausible interaction between individual mindsets and social support to predict positive behaviors.

The relation between social support and stress mindsets has been rarely studied, and existing research has yielded mixed results. In one study with Chinese healthcare professionals, Wu et al. (2024) used a social support measure, including subjective and objective support as well as participants’ uses and utilization of support, and found a moderate, positive correlation between stress mindsets and this combined social support scale. Further, they tested the mediating role of social support in the relation between empathy and stress mindsets, which was supported, indirectly demonstrating the positive correlation between social support and stress mindsets. In another study with Chinese rural-to-urban migrant children, Chen and Yang (2022) examined the interaction between stress mindsets and perceived social support via self-reports, with loneliness as the outcome, within a moderated mediation model. The results supported the moderation effect of stress mindsets: perceived social support negatively predicted loneliness, and this association was significant for migrant children holding a positive stress mindset (indicated by a high ratio of the stress-is-a-challenge mindset to the stress-is-a-threat mindset) only. This study suggests the plausible protective effects activated by the synergy of a positive stress mindset and stronger social support. Therefore, more research aimed at understanding how social support and stress mindsets work together is needed.

### 1.5. Sex Differences

Prior research suggests that sex or gender differences may be relevant in understanding how mindsets develop and operate during adolescence. Although, in this study, “sex” is used, which refers to a biological characteristic (i.e., female vs male), the relevant literature using “gender”, which is a personal and psychological characteristic determined by society, is often drawn as a distinction (e.g., boys vs. girls) to help understand the differences across two dominant groups with large overlaps in general populations. For example, early adolescent girls have been found to endorse stronger entity theories (i.e., fixed mindsets) of thoughts, feelings, and behavior than boys, and these beliefs increased over time for girls but not boys across an academic year (Schleider & Weisz, 2016). Similarly, research on beliefs about emotion indicates that girls reported stronger entity beliefs (i.e., fixed mindsets) regarding the controllability of emotions than boys, even when controlling for depressive symptoms (Ford et al., 2018). These findings suggest that gender differences in mindsets may emerge during adolescence and may have implications for developmental outcomes. However, no significant gender differences in stress mindset were found in an adult athlete sample (Mansell, 2021) or in an early adolescent sample (Ming et al., 2021). In addition, research examining the links between mindsets and psychological functioning suggests that these beliefs may operate differently across genders. For instance, entity theories (i.e., fixed mindsets) of thoughts, feelings, and behavior were more strongly associated with mental health problems for girls than for boys (Schleider & Weisz, 2016), highlighting the potential importance of considering gender when examining associations between mindset-related constructs and well-being.

Further, some studies suggest that the mechanisms linking mindsets to outcomes may differ by sex or gender. In one study of middle school students, a growth mindset predicted mathematics and science career interest indirectly through mathematics self-efficacy for boys, but this mediating pathway was not observed for girls (Huang et al., 2019). In contrast, mathematics anxiety exerted a direct influence on the career interests of girls but not boys. Research on stress mindsets has also identified gender differences in associations with well-being. Among rural-to-urban Chinese migrant adolescents, a stress-is-enhancing mindset was positively associated with life satisfaction for girls but not boys and helped to mitigate the negative effects of stressful life events on depression (Y. Jiang et al., 2019). Taken together, these findings suggest that gender may influence both the endorsement of mindset-related beliefs and the pathways through which these beliefs relate to psychological and developmental outcomes. However, research examining gender or sex differences in the mechanisms linking mindsets to developmental constructs remains limited.

When examining the mindset effect in the social context, including social support, gender socialization theory sheds important light on gender differences, which can be largely applied to group differences (by sex), broadly speaking. Early gender socialization and social learning processes are essential in shaping boys’ and girls’ senses of self and self-worth (Ruble et al., 1993) and also affect their stress reactivity and responsivity, including interpersonal orientation and coping strategies (Dedovic et al., 2009). Girls are often socialized to value social goals more and act toward interdependence, relational attunement, and support-seeking, while boys are more likely to seek independence, usually through non-social goals (e.g., achievement), and are often encouraged to behave toward autonomy and self-directed problem-solving. These differences are supported by empirical findings, such as that adolescents girls have greater sensitivity to social stress and also perceive it as more stressful than their male counterparts (Compas et al., 1985; Wagner & Compas, 1990), and girls more frequently engage in emotion-focused coping and support-seeking strategies, whereas boys more often rely on avoidant or problem-focused coping, depending on situational demands (Sigmon et al., 1995; Washburn-Ormachea et al., 2004). Moreover, gender differences exist in how social support is perceived and used in coping with stress. Girls often display greater sensitivity to interpersonal contexts and stronger reliance on relational resources for coping and adjustment (Flaherty & Richman, 1989; Matud et al., 2003), while boys may experience more constrained or ambivalent patterns of support utilization due to competing expectations around autonomy and emotional expression (Barbee et al., 1993). Within this study, stress mindsets and perceived social support represent two different types of resources: a stress mindset is largely an intrapsychic force that strengthens independence and agency, which is the primary socializing goal for boys, whereas social support is an external force built on interpersonal relationships and individuals’ accessibility or comfort to use it, which has been the emphasis in girls’ socialization. The pervasive gendered socialization processes in society likely lead to different stress mindsets and perceptions of social support between boys and girls—perhaps more so in the interaction of stress mindsets and perceived social support than just one factor alone. Therefore, further investigation is needed to determine whether and how gender differences might be present in the mechanisms involving stress mindsets, social support, and youth outcomes.

### 1.6. The Current Study

Although recent research has started to emphasize the importance of contextual influences on mindset processes, the mindset × context framework has not yet been adequately examined in the literature on stress mindsets and positive behavioral outcomes. Further, little is known about how these beliefs interact with social contexts to promote positive behavioral outcomes such as strengths use and PGI. The present study addresses this gap by examining whether perceived social support moderates the association between stress mindsets and these positive behavioral outcomes and whether this mechanism differs by sex.

Based on the theory and evidence, it is hypothesized that perceived social support will moderate the relation between stress mindsets and positive behavioral outcomes. Consistent with the mindset × context framework, the positive association between a stress-is-enhancing mindset and both personal growth initiative and strengths use is expected to be stronger at higher levels of perceived social support. At lower levels of social support, this association is expected to be weaker. Due to a lack of research on sex differences in such mechanisms, the examination of moderated moderation related to sex is exploratory, and thus no specific hypothesis is stated.

## 2. Materials and Methods

### 2.1. Participants and Procedure

This study included a total sample of 620 high school students (ages 14–19 years, *M* = 17.51 years, *SD* = 1.23). Participants were recruited as part of a larger study in spring 2022 on adolescent mindsets and mental health during the COVID-19 pandemic, approved by Temple university’s institutional review board. Data collection was completed through a Qualtrics panel. A pre-existing, cross-sectional dataset from this broader research initiative was utilized for the current study. Of these participants, 49.5% were female, 43.5% male, and 7% gender non-conforming or preferred not to answer. The racial and ethnic composition was 41% White, 17% Black or African American, 6% Asian or Asian American, 19% Hispanic or Latinx, and 3% multi-/bi-racial.

This study used an existing dataset for a larger project. The research questions and analyses are new. High school students from the South, West, Midwest, and Northeast regions of the United States participated in the study via a Qualtrics panel. This survey was administered in the spring of 2022, after in-person schooling resumed for students following the COVID-19 pandemic. Students were asked to complete an online self-report questionnaire that assessed demographic information, mindset beliefs, and positive behaviors.

### 2.2. Measures

#### 2.2.1. Stress Mindset Measure

Stress mindset was assessed using the Stress Mindset Measure (SMM; Crum et al., 2013). The scale consists of four items, where the student is asked to rate how much each statement describes them utilizing a four-point Likert scale ranging from 0 (strongly disagree) to 3 (strongly agree). Items are intended to measure the extent to which someone believes that stress is debilitating or enhancing, including items such as “The effects of stress are negative and should be avoided” and “Experiencing stress facilitates my learning and growth.” Previous research has provided data to support the adequate reliability and validity of the SMM and its utility in measuring whether individuals perceive the effects of stress as debilitating or enhancing (SMM; Crum et al., 2013). This scale showed adequate internal consistency (Cronbach’s α = 0.73).

#### 2.2.2. Social Support Measure

Social support was measured using the Social Support subscale of the Social Emotional Health Survey, Secondary School Version (SEHS-S; Furlong et al., 2014). This domain is referred to as belief-in-others and includes three subscales measuring school support, peer support, and family support (e.g., “At my school, there is a teacher or some other adult who believes that I will succeed”). The domain consists of nine items rated on a four-point Likert scale from 1 (never) to 4 (very often). Previous research supports that each domain of the SEHS-S has strong reliability and convergent validity (Furlong et al., 2014). This measure demonstrated good internal consistency (Cronbach’s α = 0.84).

#### 2.2.3. Personal Growth Initiative—Intentional Behavior (PGI-IB) Measure

PGI-IB was measured using the Intentional Behavior subscale of the Personal Growth Initiative Scale-II (PGIS-IB; Robitschek et al., 2012). The PGIS-IB was developed based on PGI theory and consists of 16 items measuring readiness for change, planfulness, and intentional behavior. The intentional behavior subscale consisted of four items, such as “I actively work to improve myself”, which were answered using a six-point Likert scale from 0 (disagree strongly) to 5 (agree strongly). The PGIS-II has strong internal consistency and acceptable convergent and discriminant validity across all subscales (Robitschek et al., 2012). This scale showed good internal consistency (Cronbach’s α = 0.89).

#### 2.2.4. Strengths Use Measure

Strengths use was measured using the Strengths Use Scale (SUS; Govindji & Linley, 2007). This scale consists of seven items measuring instances when the student utilizes their strengths or things that they do well (i.e., “I use my strengths every day”). Each item is answered on a seven-point Likert scale ranging from 1 (strongly disagree) to 7 (strongly agree). The SUS has been found to correlate with well-being and positive psychology constructs (Wood et al., 2011). This measure demonstrated good internal consistency (Cronbach’s α = 0.89).

### 2.3. Data Analysis

Descriptive, independent *t*-test (by sex group), and correlational analyses were conducted using SPSS 30.0. All core parametric assumptions were checked and met. Two multiple linear regression models were used to test the moderation hypotheses; the PROCESS macro for SPSS (Hayes, 2013) was utilized. For each model, stress mindset was the predictor, and social support was the moderator. Two separate models were conducted, with one utilizing PGI as an outcome and one using strengths use as the outcome. Due to the significant sex differences reported in the literature, additional moderated moderation models were tested to explore the role of sex in this relation. Sex was added as a second moderator along with social support, and conditional effects were estimated by looking at low, moderate, and high levels of social support at two levels of sex (male and female). There are no missing data in this dataset. It should be noted that 7% of participants did not choose male or female in the demographic section of the survey, and they were excluded from the moderated moderation models in which sex was needed for analysis. Thus, these models used a slightly smaller sample (*n* = 577). 

## 3. Results

### 3.1. Descriptives, Group Differences, and Correlations

Descriptive statistics and bivariate correlations between stress mindset, perceived social support, PGI, and strengths use are reported (see Table 1). Adolescents had an average stress mindset score of 2.54 (*SD* = 0.83, range = 1–5). On average, adolescents’ perceived score on the SEHS-S, indicating their perceived level of social support, was 2.80 (*SD* = 0.65, range = 1–4). Adolescents had an average PGI-IB score of 4.56 (*SD* = 1.04, range = 1–6) and an average strengths use score of 4.80 (*SD* = 1.17, range = 1–7). Independent *t*-test results showed significant differences in stress mindset (*t* (575) = −2.472, *p* < 0.05) between females (*M* = 2.47, *SD* = 0.79) and males (*M* = 2.65, *SD* = 0.85), but there were no significant sex differences in perceived social support (*t* (575) = −1.34, *p* = 0.18), PGI (*t* (575) = 0.921, *p* = 0.36), or strengths use (*t* (575) = −1.50, *p* = 0.14). Pearson’s correlations were used to assess the bivariate associations among stress mindset, perceived social support, PGI, and strengths use (Table 1). Stress mindset had significant low-to-moderate positive correlations with all variables. Social support was also positively correlated with PGI and strengths use.

### 3.2. One-Moderator Moderation

**Figure 1:**
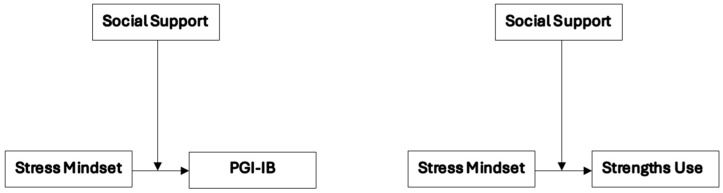
Diagram of single moderation model.

Two one-moderator moderation models (see Figure 1) were examined to determine whether social support moderated the association between stress mindset and positive behavior outcomes (see Table 2). In the single-moderator moderation model using PGI as an outcome (PGI model), no moderation effect was found, although the main effects of stress mindset and social support were both statistically significant in predicting PGI. The single moderation model using strengths use as the outcome yielded similar results (strengths use model). While there was no significant moderation effect found, there was a significant main effect of stress mindset and social support in predicting strengths use.

### 3.3. Moderated Moderations

**Figure 2:**
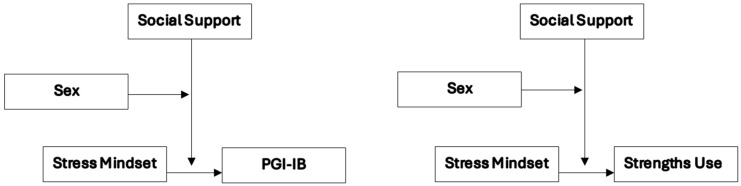
Diagram of moderated moderation models.

In the moderated moderation model examining stress mindset, social support, and PGI (see Figure 2), all interaction terms were statistically significant (Table 3 and Table 4). Most importantly, the moderation effect of social support on the relation between stress mindset and PGI was different between male and female adolescents. For males, the relation between stress mindset and PGI was strongest for groups with low social support and weaker when social support was higher. For females, while none of the conditional effects were statistically significant, the pattern was notably different from that in males: high social support groups had the strongest association between stress mindset and PGI, and this relation was weakened as social support declined.

The moderated moderation model examining strengths use as an outcome yielded similar results. All interaction terms were statistically significant. Specifically, the magnitude of the positive relation between stress mindset and strengths use was weaker at higher levels of social support among males; this relation was stronger when the social support level was high for females, showing the opposite trend. Across sexes, groups with higher social support always reported higher PGI and strengths use, regardless of the level of stress mindset.

## 4. Discussion

The current study aimed to explore the associations between stress mindsets, perceived social support, and positive behavioral outcomes (PGI and strengths use) through moderation models. Given the past literature suggesting that sex differences are present in terms of mindset, this study also aimed to explore whether there were any differences within this study specifically related to sex. It was found that stress mindset was positively related to all other variables, suggesting that adolescents who endorse more of a stress-is-enhancing mindset are more likely to intentionally engage in personal growth, while individuals with a stress-is-debilitating mindset were typically found to display fewer of these behaviors. It was also found that perceived social support and sex moderated the relations between stress mindset and positive behavioral outcomes for both the PGI and strengths use models. Further, the results revealed different patterns in the relations between stress mindset and positive behavioral outcomes between males and females. Overall, these findings provide partial support for the study hypotheses, indicating that perceived social support does moderate the relationship between stress mindsets and positive behavioral outcomes; however, the direction of this effect varied by sex and did not uniformly align with the expected pattern.

First, in this sample, there was a significant difference in the stress mindset levels between male and female adolescents, with male adolescents reporting more growth-oriented stress mindsets and female adolescents endorsing stronger fixed stress mindsets. This is consistent with the broader mindset research literature that suggests stronger fixed-oriented mindsets in female adolescents (e.g., implicit theories of thoughts, feelings, and behavior, Schleider & Weisz, 2016; mindsets about emotions, Ford et al., 2018). Regarding stress mindset research in particular, the results are mixed across a few divergent samples (e.g., no significant difference in mid-aged adult athletes, Mansell, 2021; no correlation between stress mindset and gender among an early adolescent sample in China, Ming et al., 2021). The current study adds to the literature showing that, likely among middle–late adolescents in the United States, male adolescents’ self-reporting of stress mindsets is more positive than that of their female counterparts.

Second, the most notable finding of this study was the significant moderated moderation effect, indicating that the association between stress mindset and positive behavioral outcomes depended on both perceived social support and adolescent sex. The significant moderating effect of stress mindset in the model is consistent with a previous study with an adolescent sample (Chen & Yang, 2022), which found that stress mindset moderated the relation between perceived social support and loneliness in Chinese rural-to-urban migrant youth (i.e., the negative association between social support and loneliness was significant only for those with a positive stress mindset). These findings together provide further support for the mindset × context framework by demonstrating that the effects of stress mindsets depend on social and environmental conditions.

Further, the present study also added another layer to the mindset × context framework by revealing the significant individual differences by sex. Consistent with the mindset × context framework, perceived social support functioned as a significant moderator; however, the hypothesized pattern (i.e., stronger positive associations were expected at higher levels of support) was only evident among female adolescents, whereas an inverse pattern emerged for males. Specifically, the results indicated that the relation between a stress-is-enhancing mindset and positive behavioral outcomes varied as a function of perceived social support in different ways for males and females. For males, the association between stress mindset and both personal growth initiative and strengths use was strongest under conditions of low social support, and the association weakened as social support increased. In contrast, for females, the pattern was reversed; the association between stress mindset and positive behavioral outcomes was strongest at higher levels of social support and weaker at lower levels, although conditional effects were not statistically significant for this group. As no specific hypotheses were proposed regarding sex differences, these findings offer novel insight into how these processes may differ across groups.

These findings can be understood in the context of the broader literature on sex differences in stress coping and gender socialization processes. First, the overall finding of the model differences supports established scientific evidence on different stress reactivity between males and females that is partially explained by biological differences (e.g., stress hormones; for a review, see Dedovic et al., 2009). Second, the three-way interactions between stress mindset, perceived social support, and sex reveal phenomena beyond just sex differences but suggest the necessity of considering gender (i.e., social and cultural differences between boys and girls) in explaining the observed differences in stress responses between boys and girls. Specifically, perceived social support in the environment appears to play an important role in how stress-related beliefs are translated into behavior for boys and girls. For girls, higher perceived social support seems to provide fertile soil for growing a more positive stress mindset, which can lead to more positive behaviors. This finding corresponds to gender socialization’s impact on girls: social and cultural norms, expectations, and daily routines infuse and reinforce the importance of connectedness with others, and the perception of social connectedness plays a vital role in girls’ stress reactivity, including to what extent a positive stress mindset can increase agentic behaviors. In contrast, higher social support does not bring the same amplifying effect to boost the benefits of stress mindsets for boys as for girls. When perceiving high social support, the influence of boys’ stress mindsets on their active and intentional involvement in their personal development or use of strengths appears to operate independently of the available social support. Interestingly, when boys perceive lower social support, the benefit of having a more positive stress mindset becomes more salient, possibly through activating greater agency in the absence of adequate external relational resources that lead to more agentic behavior, such as personal growth initiative and the use of their own strengths. This pattern is consistent with socialization processes for boys that emphasize independence and self-reliance. It is important, however, to acknowledge that no causal claims can be made regarding gender due to the correlational nature of this study. Previous research has found that social support seeking may be influenced by prior emotion socialization, such as emotional support from a caregiver when distressed (Marousis & Luebbe, 2025). It is possible that an adolescent’s previous experience with social support offered has an impact on their perception of it, regardless of gender. Together, these patterns appear to indicate that the pathway from a stress mindset to behavior is not only context-dependent but also potentially shaped by gendered socialization processes that influence how adolescents access and utilize social support in stressful contexts.

Despite the sex differences, it should be noted that, across female and male adolescents, higher perceived social support was consistently associated with higher levels of positive behavioral outcomes, regardless of the stress mindset. This reinforces the notion that social support is critical and beneficial for adolescents’ positive behavior development. In addition, while similar conditional effects were found related to sex when looking at both moderation models, there were slight differences between the two positive behavioral outcomes examined. The effects of social support were seen to be slightly stronger when using strengths use as an outcome for females. The association between a stress-is-enhancing mindset and positive behavior outcomes in general was strongest at high levels of social support for female adolescents. In the model with strengths use as the outcome, the effect of social support nearly doubled. These differences indicate that social support may have a stronger effect on female adolescents’ use of their strengths. Overall, higher perceived social support was associated with positive behavioral outcomes across both sexes, regardless of the stress mindset, emphasizing the importance of social support for adolescents.

### 4.1. Limitations and Future Research

While this study yielded significant and novel results, there are several limitations that must be considered and can help inform future research on this topic. The current study utilized data from a pre-existing dataset collected in 2022. The study also used a cross-sectional design; therefore, temporal ordering among variables cannot be established, and no inferences can be made regarding the directionality or causality of the relationships. Because all data were collected at a single point in time, the results cannot be used to predict longitudinal relations. Further, these data were collected closely following a global health crisis, which might have had an impact on adolescents’ mindsets and behaviors at the time, and the results might not generalize to different contexts. All data were also collected using self-reports only, being subject to single-method bias. Given the self-report nature of this study, social support was only measured as an individual perception rather than a concrete resource.

Future research should seek to further explore the associations between stress mindsets, perceived social support, and positive behavior outcomes in adolescents. These associations should also be explored in samples representing different developmental ages or other characteristics (e.g., at-risk and clinical populations), as well as populations with limited access to resources, lower socioeconomic status, and/or other risk factors for youth development (e.g., homelessness, poverty, dysfunctional families, health issues or disability). It is worthwhile to examine the institutional and community support systems in place for adolescents to utilize. Moreover, since the focus is on the interplay of individual mindsets and contextual factors, broader cultures or cultural dimensions (e.g., individualism vs. collectivism, competition and support in educational environments) likely influence such mechanisms. Relatedly, more work is needed to explore cultural and other contextual factors that may affect the mechanisms, such as social norms, economic systems, religion, and media influences.

Methodology-wise, longitudinal data will be needed to examine whether the observed sex-specific moderation patterns reflect developmental traits that change over time or represent more contextually stable differences. Longitudinal findings would also provide more evidence to guide stress mindset-based interventions. Future studies should also take into account multiple methods of measurement, such as parent and teacher ratings of adolescents’ use of positive behaviors or how they support adolescents beyond self-report measures. As this study only focused on two positive behavioral outcomes, future research should also aim to examine other positive outcomes that may be associated with a stress-is-enhancing mindset and social support. Further, the differences in how males and females perceive and utilize this support should be studied in more detail, given that this study’s results regarding sex differences were exploratory.

### 4.2. Implications

Importantly, the present study does not directly examine intervention design, implementation processes, or the comparative effectiveness of specific methods of modifying stress mindsets. As such, the implications should be interpreted as theoretically informed applications. The findings instead identify potential contextual conditions, particularly perceived social support and sex-related differences, that appear to shape how a stress mindset relates to positive behavioral outcomes. This provides a basis for generating hypotheses about where and for whom stress mindset processes may be most relevant; however, more targeted experimental and longitudinal research is needed to establish how such mechanisms can be effectively translated into intervention approaches.

The results suggest that efforts to promote stress-is-enhancing mindsets may be most impactful when embedded within adolescents’ existing social environments, as schools, caregivers, and community-based programs can serve as natural contexts in which messages about stress can be communicated and reinforced, particularly given the role of perceived social support observed in this study. The effectiveness of such efforts may differ depending on individual and contextual factors, including sex and the level of support available, so future research is needed to identify the best approaches for targeting stress mindsets and to determine which sources of social influence are most effective in promoting adaptive beliefs about stress among adolescents. In particular, resilience can be understood as a process that is shaped by both individual beliefs and the social contexts in which those beliefs are enacted. Across school, family, and community contexts, intervention developers and service providers should adopt a more integrated approach that targets both adolescents’ beliefs about stress and the broader interpersonal environments, including institutional and community support structures, in which they are embedded. In addition, sex differences should be considered in terms of how individual students may perform or grow positive behaviors differently, depending on the combination of their own stress beliefs and support perceived in the external environment. For example, social support tends to play a more facilitative role for female adolescents that amplifies the positive effect of a growth-oriented stress mindset, while male adolescents appear to rely more heavily on their internal beliefs about stress in the absence of such support. Thus, interventions for female adolescents may be strengthened by explicitly leveraging relational contexts to reinforce adaptive stress beliefs, whereas, for male adolescents, greater emphasis may be placed on building internal coping frameworks, alongside increasing comfort with being supported externally without diminishing their internal strengths. Altogether, this study highlights the importance of understanding the mindset × context framework, along with accounting for sex and the influence of gender socialization, to promote resilience among adolescents.

## Figures and Tables

**Table 1:** Means, standard deviations, and bivariate correlations.

	*M*	*SD*	1	2	3	4
1. Stress mindset	2.54	0.83	-	−0.13 **	0.12 **	0.22 **
2. Social support	2.80	0.65	-	-	0.33 **	0.40 **
3. PGI-IB	4.56	1.04	-	-		0.53 **
4. Strengths use	4.80	1.17	-	-	-	-

*Note*: ** *p* < 0.01.

**Table 2:** Results of single moderation models.

PGI Model	*R*	*R-Sq*	*F*	*p*	*df1*	*df2*
Model summary	0.35 **	0.12	27.95	0.00	3	616
	*coeff*	*se*	*t*	*p*	*LLCI*	*ULCI*
Constant	2.15 **	0.53	4.04	0.00	1.10	3.20
Stress mindset	0.39	0.21	1.89	0.06	−0.02	0.79
Social support	0.78 **	0.19	4.17	0.00	0.41	1.14
Stress mindset × social support	−0.11	0.07	−1.48	0.14	−0.24	0.03
Strengths use model	*R*	*R-sq*	*F*	*p*	*df1*	*df2*
Model summary	0.43 **	0.19	47.54	0.00	3	616
Constant	1.87 **	0.58	3.24	0.001	0.74	2.99
Stress mindset	0.41	0.22	1.85	0.06	−0.02	0.85
Social support	0.84 **	0.20	4.14	0.00	0.44	1.23
Stress mindset × social support	−0.06	0.08	−0.83	0.41	−0.21	0.09

*Note*: ** *p* < 0.01.

**Table 3:** Results of moderated moderation model with personal growth initiative (PGI) as the outcome.

PGI Model		*R*	*R-Sq*	*F*	*p*	*df1*	*df2*
Model summary		0.37	0.13	12.68	0.00	7	569
		*coeff*	*se*	*t*	*p*	*LLCI*	*ULCI*
Constant		7.6 **	1.86	4.09	0.00	3.95	11.25
Stress mindset		−1.53 *	0.73	−2.10	0.04	−2.97	−0.10
Social support		−1.01	0.65	−1.53	0.13	−2.29	0.28
Sex		−3.66 **	1.16	−3.15	0.002	−5.95	−1.38
Stress mindset × social support		0.54 *	0.25	2.11	0.04	0.04	1.04
Stress mindset × sex		1.28 *	0.45	2.84	0.01	0.40	2.17
Social support × sex		1.18 **	0.41	2.89	0.004	0.38	1.97
Stress mindset × social support × sex		−0.42 *	0.16	−2.71	0.01	−0.72	−0.12
Test of highest-order unconditional interactions			*R-sq*	*F*	*p*	*df1*	*df2*
Stress mindset × social support × sex			0.01 *	7.35	0.01	1	569
Test of condition second-highest interaction at values of sex			*effect*	*F*	*p*	*df1*	*df2*
Female			0.19	1.03	0.31	1	569
Male			0.30 **	8.76	0.003	1	569
Conditional effect based on sex		*effect*	*se*	*t*	*p*	*LLCI*	*ULCI* level of social support
Female	Low: 25th percentile	0.01	0.10	0.04	0.97	−0.19	0.20
	Medium: 50th percentile	0.08	0.07	1.12	0.26	0.06	0.22
	High: 75th percentile	0.16	0.12	1.46	0.14	0.05	0.36
Male	Low: 25th percentile	0.37 **	0.11	3.59	0.00	0.17	0.58
	Medium: 50th percentile	0.18 *	0.07	2.47	0.01	0.04	0.32
	High: 75th percentile	−0.01	0.09	−0.16	0.88	−0.19	0.16

*Note*: * *p* < 0.05; ** *p* < 0.01.

**Table 4:** Results of moderated moderation model with strengths use as the outcome.

Strengths Use Model		*R*	*R-Sq*	*F*	*p*	*df1*	*df2*
Model summary		0.43 **	0.19	47.54	0.00	3	616
		*coeff*	*se*	*t*	*p*	*LLCI*	*ULCI*
Constant		7.11 **	1.96	3.63	0.00	3.27	10.95
Stress mindset		−1.54 *	0.77	−2.00	0.05	−3.05	0.03
Social support		−1.0	0.68	−1.45	0.15	−2.35	0.36
Sex		−3.16 *	1.22	−2.59	0.01	−5.57	−0.76
Stress mindset × social support		0.60	0.27	2.25	0.25	0.08	1.13
Stress mindset × sex		1.18 *	0.47	2.49	0.01	0.25	2.11
Social support × sex		1.14 *	0.43	2.67	0.01	0.30	1.98
Stress mindset × social support × sex		−0.41 *	0.16	−2.53	0.01	−0.73	−0.09
Test of highest-order unconditional interactions			*R-sq*	*F*	*p*	*df1*	*df2*
Stress mindset × social support × sex			0.01 *	6.41	0.01	1	569
Test of condition second-highest interaction at values of sex			*effect*	*F*	*p*	*df1*	*df2*
Female			0.19	2.4	0.12	1	569
Male			0.22*	4.31	0.04	1	569
Conditional effect based on sex and level of social support		*effect*	*se*	*t*	*p*	*LLCI*	*ULCI*
Female	Low: 25th percentile	0.04	0.11	0.37	0.71	−0.18	0.26
	Medium: 50th percentile	0.19 *	0.08	2.48	0.01	0.04	0.34
	High: 75th percentile	0.29 *	0.11	2.66	0.01	0.08	0.51
Male	Low: 25th percentile	0.35 **	0.11	3.05	0.002	0.12	0.57
	Medium: 50th percentile	0.17 *	0.08	2.3	0.02	0.03	0.32
	High: 75th percentile	0.05	0.10	0.54	0.59	−0.14	0.24

*Note:* * *p* < 0.05; ** *p* < 0.01.

## Data Availability

The data presented in this study are available on request from the corresponding author.

## Abbreviations

The following abbreviations are used in this manuscript:

PGI	Personal growth initiative
PGI-IB	Personal growth initiative—intentional behavior
